# Supplementary material for: MEME-LaB: motif analysis in clusters
Source: Bioinformatics. 2013 May 14;29(13):1696–7. doi: 10.1093/bioinformatics/btt248 (PMC3694638; doi:10.1093/bioinformatics/btt248)
Supplement: Supplementary Data [file supp_btt248_Supplementary_Data.zip › Supplementary_Data/Results_Files/which_gene_ids/cluster18_motif1.html]

Cluster 18 Motif 1


Cluster 18 Motif 1 is found in the following promoters

| Gene ID | Strand | Position\* |
| --- | --- | --- |

|  |  |  |
| --- | --- | --- |
| AT1G01840 | - | 95/500 |
| AT1G02120 | - | 484/500 |
| AT1G02840 | - | 370/500 |
| AT1G03475 | + | 58/500 |
| AT1G04710 | + | 331/500 |
| AT1G05670 | + | 148/500 |
| AT1G05970 | + | 213/500 |
| AT1G06900 | + | 328/500 |
| AT1G07880 | - | 274/500 |
| AT1G08460 | - | 404/500 |
| AT1G08760 | - | 363/500 |
| AT1G09100 | + | 307/500 |
| AT1G09440 | + | 411/500 |
| AT1G09530 | + | 379/500 |
| AT1G09820 | + | 417/500 |
| AT1G11755 | - | 194/500 |
| AT1G13570 | - | 162/500 |
| AT1G14590 | - | 81/500 |
| AT1G15215 | + | 97/500 |
| AT1G17110 | + | 126/500 |
| AT1G17480 | - | 447/500 |
| AT1G17580 | - | 197/500 |
| AT1G17820 | + | 416/500 |
| AT1G18335 | - | 333/500 |
| AT1G19400 | - | 203/500 |
| AT1G19570 | + | 180/500 |
| AT1G20560 | + | 348/500 |
| AT1G23190 | + | 410/500 |
| AT1G26550 | - | 353/500 |
| AT1G27960 | + | 439/500 |
| AT1G31150 | - | 394/500 |
| AT1G31400 | - | 216/500 |
| AT1G32610 | + | 220/500 |
| AT1G35780 | + | 180/500 |
| AT1G42430 | + | 368/500 |
| AT1G43700 | - | 197/500 |
| AT1G44100 | - | 113/500 |
| AT1G50120 | + | 85/500 |
| AT1G51965 | + | 321/500 |
| AT1G53310 | + | 359/500 |
| AT1G57540 | + | 349/500 |
| AT1G59520 | + | 445/500 |
| AT1G60690 | + | 469/500 |
| AT1G63700 | - | 438/500 |
| AT1G64690 | - | 473/500 |
| AT1G65930 | - | 160/500 |
| AT1G67950 | - | 438/500 |
| AT1G67960 | - | 488/500 |
| AT1G68720 | - | 57/500 |
| AT1G68920 | + | 340/500 |
| AT1G70470 | + | 390/500 |
| AT1G70570 | + | 6/500 |
| AT1G73020 | + | 172/500 |
| AT1G73100 | + | 288/500 |
| AT1G73150 | + | 29/500 |
| AT1G74260 | - | 78/500 |
| AT1G78960 | + | 37/500 |
| AT1G80060 | - | 197/500 |
| AT1G80340 | + | 306/500 |
| AT2G01350 | - | 476/500 |
| AT2G02070 | - | 481/500 |
| AT2G06095 | + | 119/500 |
| AT2G07779 | + | 406/500 |
| AT2G16960 | - | 411/500 |
| AT2G17990 | - | 106/500 |
| AT2G18960 | + | 329/500 |
| AT2G19400 | - | 372/500 |
| AT2G20875 | - | 162/500 |
| AT2G21070 | - | 179/500 |
| AT2G22200 | + | 472/500 |
| AT2G22490 | - | 333/500 |
| AT2G22840 | - | 464/500 |
| AT2G23070 | + | 445/500 |
| AT2G24720 | + | 482/500 |
| AT2G25280 | + | 396/500 |
| AT2G25310 | + | 390/500 |
| AT2G25530 | - | 391/500 |
| AT2G25790 | - | 390/500 |
| AT2G25910 | - | 458/500 |
| AT2G26350 | + | 242/500 |
| AT2G28080 | - | 451/500 |
| AT2G31440 | + | 424/500 |
| AT2G32080 | + | 49/500 |
| AT2G32810 | - | 67/500 |
| AT2G33620 | + | 157/500 |
| AT2G33630 | - | 150/500 |
| AT2G34640 | + | 321/500 |
| AT2G34710 | - | 105/500 |
| AT2G35010 | + | 146/500 |
| AT2G35320 | + | 381/500 |
| AT2G35800 | + | 482/500 |
| AT2G36530 | + | 117/500 |
| AT2G36570 | - | 422/500 |
| AT2G37200 | + | 87/500 |
| AT2G38070 | + | 153/500 |
| AT2G39180 | - | 13/500 |
| AT2G39750 | - | 373/500 |
| AT2G41020 | - | 74/500 |
| AT2G41220 | - | 91/500 |
| AT2G42290 | - | 147/500 |
| AT2G42680 | + | 2/500 |
| AT2G42700 | + | 140/500 |
| AT2G43370 | + | 287/500 |
| AT2G45120 | + | 26/500 |
| AT2G46640 | + | 241/500 |
| AT2G46800 | + | 375/500 |
| AT2G47010 | + | 217/500 |
| AT2G47420 | + | 453/500 |
| AT3G03100 | - | 140/500 |
| AT3G03330 | - | 324/500 |
| AT3G03520 | + | 9/500 |
| AT3G03890 | - | 141/500 |
| AT3G04590 | + | 458/500 |
| AT3G04610 | + | 198/500 |
| AT3G04740 | - | 482/500 |
| AT3G04950 | + | 368/500 |
| AT3G06110 | + | 115/500 |
| AT3G06200 | - | 163/500 |
| AT3G06240 | + | 155/500 |
| AT3G06290 | + | 142/500 |
| AT3G06790 | + | 51/500 |
| AT3G06850 | - | 443/500 |
| AT3G07060 | + | 202/500 |
| AT3G07540 | - | 178/500 |
| AT3G07790 | - | 23/500 |
| AT3G08850 | + | 434/500 |
| AT3G09470 | - | 134/500 |
| AT3G10920 | + | 54/500 |
| AT3G12750 | - | 450/500 |
| AT3G13350 | - | 49/500 |
| AT3G14940 | + | 310/500 |
| AT3G15290 | - | 207/500 |
| AT3G16190 | - | 183/500 |
| AT3G17020 | - | 264/500 |
| AT3G17940 | - | 96/500 |
| AT3G18215 | + | 111/500 |
| AT3G19120 | - | 469/500 |
| AT3G19190 | + | 53/500 |
| AT3G19990 | - | 431/500 |
| AT3G22270 | - | 223/500 |
| AT3G23090 | + | 194/500 |
| AT3G26935 | - | 458/500 |
| AT3G27220 | + | 398/500 |
| AT3G42630 | + | 386/500 |
| AT3G42660 | + | 354/500 |
| AT3G43580 | + | 405/500 |
| AT3G43600 | - | 385/500 |
| AT3G43790 | - | 265/500 |
| AT3G44220 | + | 281/500 |
| AT3G48170 | + | 342/500 |
| AT3G48210 | + | 40/500 |
| AT3G48740 | - | 417/500 |
| AT3G48810 | + | 287/500 |
| AT3G49600 | - | 196/500 |
| AT3G50670 | + | 375/500 |
| AT3G51610 | - | 323/500 |
| AT3G52090 | + | 231/500 |
| AT3G52880 | + | 200/500 |
| AT3G53020 | - | 202/500 |
| AT3G54140 | - | 51/500 |
| AT3G55160 | - | 473/500 |
| AT3G56150 | - | 87/500 |
| AT3G57060 | - | 193/500 |
| AT3G57610 | + | 82/500 |
| AT3G57790 | + | 419/500 |
| AT3G58600 | + | 51/500 |
| AT3G60740 | + | 214/500 |
| AT3G60850 | + | 455/500 |
| AT3G62290 | + | 15/500 |
| AT3G62860 | + | 485/500 |
| AT4G01000 | + | 231/500 |
| AT4G05530 | + | 117/500 |
| AT4G08460 | + | 393/500 |
| AT4G08850 | - | 37/500 |
| AT4G12050 | + | 484/500 |
| AT4G12300 | + | 468/500 |
| AT4G12640 | + | 440/500 |
| AT4G14880 | + | 434/500 |
| AT4G15840 | + | 145/500 |
| AT4G16130 | + | 455/500 |
| AT4G16570 | - | 131/500 |
| AT4G17280 | - | 269/500 |
| AT4G20350 | + | 158/500 |
| AT4G20360 | - | 444/500 |
| AT4G21770 | - | 149/500 |
| AT4G22930 | - | 489/500 |
| AT4G23410 | - | 405/500 |
| AT4G23440 | - | 410/500 |
| AT4G24680 | + | 374/500 |
| AT4G25370 | + | 172/500 |
| AT4G25500 | + | 452/500 |
| AT4G27990 | + | 52/500 |
| AT4G28070 | + | 330/500 |
| AT4G28650 | - | 142/500 |
| AT4G30550 | + | 329/500 |
| AT4G31160 | + | 193/500 |
| AT4G32180 | - | 367/500 |
| AT4G33410 | + | 114/500 |
| AT4G33530 | - | 489/500 |
| AT4G34040 | + | 330/500 |
| AT4G34070 | + | 179/500 |
| AT4G34270 | - | 453/500 |
| AT4G35140 | - | 472/500 |
| AT4G36195 | - | 94/500 |
| AT4G36630 | - | 457/500 |
| AT4G37130 | + | 28/500 |
| AT4G38500 | + | 187/500 |
| AT4G38520 | - | 452/500 |
| AT4G38670 | + | 473/500 |
| AT4G38730 | - | 488/500 |
| AT4G38800 | - | 203/500 |
| AT4G39730 | + | 262/500 |
| AT5G02480 | + | 343/500 |
| AT5G04750 | + | 256/500 |
| AT5G05180 | + | 374/500 |
| AT5G05660 | - | 426/500 |
| AT5G05710 | + | 110/500 |
| AT5G07610 | - | 67/500 |
| AT5G08100 | + | 438/500 |
| AT5G09540 | + | 249/500 |
| AT5G11510 | + | 441/500 |
| AT5G11810 | - | 77/500 |
| AT5G12120 | + | 359/500 |
| AT5G12840 | + | 117/500 |
| AT5G13520 | - | 88/500 |
| AT5G14510 | + | 255/500 |
| AT5G15270 | + | 155/500 |
| AT5G15680 | + | 432/500 |
| AT5G16040 | - | 477/500 |
| AT5G16050 | - | 449/500 |
| AT5G16310 | + | 121/500 |
| AT5G16810 | - | 468/500 |
| AT5G17160 | - | 311/500 |
| AT5G18040 | + | 228/500 |
| AT5G20510 | - | 101/500 |
| AT5G24310 | + | 481/500 |
| AT5G24830 | + | 363/500 |
| AT5G24890 | + | 342/500 |
| AT5G27606 | - | 400/500 |
| AT5G28010 | + | 285/500 |
| AT5G35360 | + | 394/500 |
| AT5G37560 | + | 195/500 |
| AT5G42220 | - | 466/500 |
| AT5G42470 | + | 410/500 |
| AT5G43330 | + | 275/500 |
| AT5G48230 | - | 389/500 |
| AT5G49215 | + | 334/500 |
| AT5G49980 | + | 429/500 |
| AT5G50340 | + | 489/500 |
| AT5G52547 | - | 140/500 |
| AT5G52840 | + | 246/500 |
| AT5G53350 | - | 315/500 |
| AT5G53400 | - | 273/500 |
| AT5G54280 | + | 471/500 |
| AT5G54880 | + | 474/500 |
| AT5G55670 | + | 299/500 |
| AT5G55900 | - | 104/500 |
| AT5G58490 | + | 367/500 |
| AT5G58690 | + | 203/500 |
| AT5G58960 | - | 433/500 |
| AT5G62090 | - | 450/500 |
| AT5G62370 | + | 93/500 |
| AT5G62820 | + | 482/500 |
| AT5G62960 | - | 36/500 |
| AT5G64350 | - | 240/500 |
| AT5G64360 | - | 456/500 |
| AT5G65960 | - | 240/500 |
| AT5G66300 | - | 431/500 |
| AT5G66510 | + | 88/500 |

**\*** Overlapping sequences are merged to avoid redundancy. As a result some sequences in the final set may be longer than the specified maximum.

Distribution

+

-

Position values represent number of bases from the 5 prime end of the sequence provided to MEME and the length of that sequence. It is these figures expressed as proportions that are used for statistical analysis.

Probability value of comparison of these values to a linear distribution, p = 9.6e-05

The motif is found 151 times on the + stand and 118 times on the - strand.

Probability value that it is evenly distributed between the two strands, p = 0.025

Move the mouse pointer over the gene ids to highlight the corresponding marker
